# Prenatal screening tests may be a warning for the partial molar pregnancy? case report

**DOI:** 10.11604/pamj.2015.20.323.5995

**Published:** 2015-04-06

**Authors:** Mehmet Akif Sargin, Niyazi Tug, Murat Yassa, Arzu Yavuz

**Affiliations:** 1 Department of Obstetrics and Gynecology, Fatih Sultan Mehmet Research and Training Hospital, Istanbul, Turkey; 2 Department of Obstetrics and Gynecology, Derince Research and Training Hospital, Kocaeli, Turkey

**Keywords:** Partial hydatidiform mole, complet hydatidiform, prenatal screening

## Abstract

Prenatal screening tests are frequently requested for chromosomal abnormalities. Placental pathologies in early pregnancy may be overlooked, especially in partial molar pregnancy. We are reporting an incorrect preliminary diagnosed case with an increased risk of Down syndrome in her first-trimester screening test due to partial molar pregnancy.

## Introduction

Hydatidiform moles are the result of abnormal fertilization with the incidence about one in 500-1000 pregnancies. Hydatidiform mole is the most common form of gestational trophoblastic disease (GTD). Pathological and cytogenetic studies have demonstrated that molar pregnancies may be complete, partial or invasive. Partial mole results from the fertilization of an ovum by two sperms. So usually triploid embryonal (about 90 percent) formation occurs with the features of the chorionic villi and trophoblastic hyperplasia [1]. Serum B-hcg levels are rarely bigger than 100,000 mIU/mL. Complete moles (CHM) results from the fertilization of an empty egg with a haploid sperm. Most commonly, Complete moles have a 46,XX karyotype that formed by paternal origin. Fetal tissue demonstrates minimal embryonal development with diffuse swelling of the chorionic villi and diffuses trophoblastic hyperplasia [2]. Considering the gestational age, serum B-hcg levels are significantly more elevated than it should be. Obstetric complications such as hyperemesis gravidarum, preeclampsia before 20 weeks of gestation and hyperthyroidism are more common in complete mole than partial mole. Invasive hydatidiform mole, also known as chorioadenoma destruens is a type of neoplasia that penetrates into the myometrium, vascular spaces or extrauterine areas. Definitive diagnosis made by evacuation of the uterine contents by suction curettage and histological examination of tissue [3].

## Patient and observation

**Figure 1:**
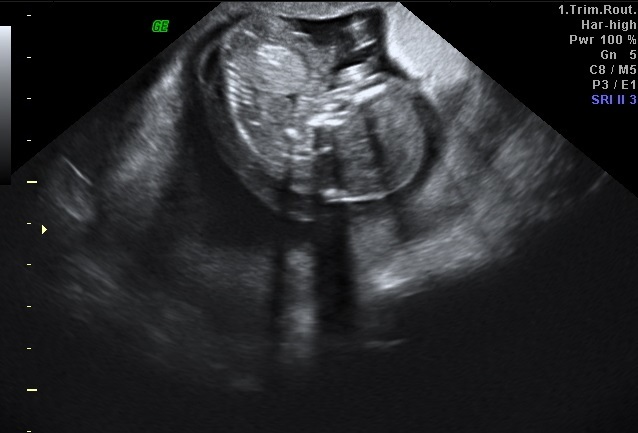
Fetus with septad cystic hygroma and hydrops fetalis

**Figure 2:**
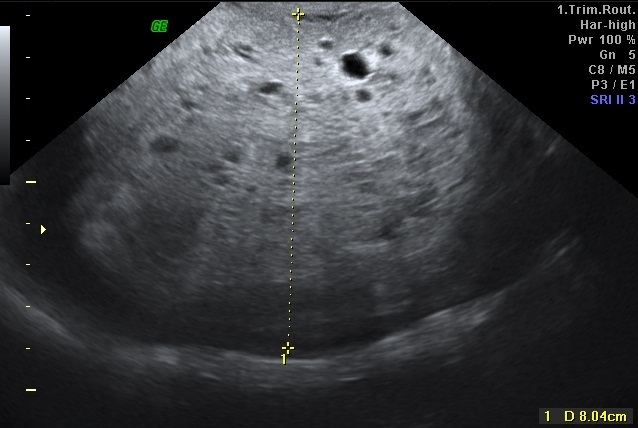
The thick swiss cheese pattern placenta

A 27-year-old gravida 2, para 1 woman was referred to our hospital for further obstetric examination of her first-trimester screening test which was associated with an increased risk of Down syndrome. Her menstrual periods were regular, the first day of her last menstrual period was 14 weeks ago. The first trimester screening test value of B-hcg was 4, 3 MoM and correspondingly to this result, Down syndrome risk had been found 1/80. Transvaginal ultrasound examination showed a CRL 13 weeks plus 6 days alive fetus with septad cystic hygroma and hydrops fetalis (Figure 1). The thick Swiss-Cheese pattern placenta (Figure 2) and normal adnexa revealed. The preliminary diagnosis was considered as partial molar pregnancy. Patient was normotensive. In laboratory, serum B-hcg 550000 IU/ml, Hgb 12 gr/dl, decreased TSH and negatif proteinuria in urinanalysis found. There was no hyperthyroidism. Chest X-ray was unremarkable. Pregnancy termination and suction curetage planned under general anesthesia due to the fetal anomaly and prediagnosis of partial molar pregnancy. Oral misoprostol treatment began once every four hours. After abortion, suction curettage was performed. Patient was discharged on the first postoperative day. Histopathological examination results indicated partial molar pregnancy. Karyotype analysis could not be performed because the placenta was received fixed in formalin. Patient was followed up for 48 hours after the evacuation and weekly until serum B-hcg has fallen to undetectable levels. Twelfth week B-hcg value was negative. Once undetectable level was attained, follow up was continued monthly for an additional 9 months. The mean follow up period of the patient's was one year.

## Discussion

Partial and complete moles are differentiated by distinct in karyotype, gross morphology, histologic characteristics, and clinical features. Obstetric complications such as hyperemesis, preeclampsia, hypertyroid and theca lutein cysts are most common in complete mole than partial mole due to very high serum B-hcg levels. Normal pregnancy peak B-hcg level is typically 100,000 mIU/mL) [4]. Patients with PHM rarely present with markedly eleveted hcg levels. In literature review about partial mol and persistant gestastional trofoblastic disease markedly elevated B-hcg levels had been reported numerously [5]. Extremely elevated levels (>2,000,000 mIU/mL) had been reported as well [5, 6]. Fetal anomaly was not reported in those cases. Our patient's calculated B-hcg level was 550,000 mIU/mL and fetal anomalies (septad cystic hygroma and hydrops fetalis) were seen by transvaginal ultrasonography exam. Clinical presentation of GTD may include vaginal bleeding, enlarged uterus, pelvic pressure or pain. Occasionally GTD can be diagnosed after obstetric complications such as theca lutein cysts, hyperemesis gravidarum, hyperthyroidism and early preeclampsia. Our patient had only subclinical hyperthyroidism among the symptoms mentioned above. Patient has been referred to our clinic for routine pregnancy care and the high risk of Down Syndrome in the first trimester screening. Rise in the risk was due to the high B-hcg levels. Beside demonstrating the fetal anomaly by ultrasonographically, placental appearance was matched with molar pregnancy in our patient. We conclusively diagnosed the molar pregnancy by histopathological assessment after the termination of pregnancy. While evaluating the first trimester screening tests, obstetricians should be alert for molar pregnancy upon encountering the high B-hcg MoM levels. Ultrasonographic image of partial mol pregnancy may not always be typical. Quantitative B-hcg measurements should be repeated if necessary. Postpartum histopathological study for partial molar pregnancy shall be performed for whom analysis showed diploid karyotype with high B-hcg Mom levels and who does not want any further karyotype testing. There have been reported more than 200 cases of twin pregnancy with CHM and a coexisting normal fetus in literature, while more than 56 cases result in live birth [7]. The fetal or embryonic tissue that present with partial mole diagnosis will most commonly have triploid karyotype (about 90 percent). However, Lage JM et al reported that flow cytometry studies for partial mole have revealed a variety of other karyotypes in the remaining 10 percent [8]. E. Jauniaux et al reported PMP that early diagnosed by ultrasonography and confirmed with postpartum histopathological examination of placenta. They reported two healthy babies delivered by vaginally at 39^th^ and 40^th^ weeks of pregnancy with normal karyotype [9]. Genest DR et al contradict to the presence of partial molar pregnancy with diploid karyotype [1]. They suggest careful reevaluation of putative specimens to uncover pathologic or ploid errors.

## Conclusion

In summary, we recommend to keep in mind that partial mole could exist in pregnancies whom B-Hcg MoM values are high in the prenatal screening tests. Routine monitoring for development of postmolar GTN by weekly hCG levels measurement until acquiring three consecutive normal values or postpartum routine pathological evaluation of the placenta should be done. Thus, earliest detection of low-risk GTN patients would be possible by determining the high-risk antecedent pregnancy cases (term, scored 2) and interval months from index pregnancy (
